# 

**Published:** 1888-01

**Authors:** 


### CONTENTS.

### COMMUNICATIONS.

Protective Dentine, or Dentine of Repair........ 5
Breathing Pure Oxygen........ 17
Some Suggestions on Metal Cap Crowns........ 17
Near Approach to, and Devitalization of the Dental Pulp........ 23
Ammonia vs. Zinc Fillings........ 25
Reflex(ions)........ 27
Tin and Gold as a Filling........ 31
Extraction of Teeth........ 32
Pyorrhœa Alveolaris........ 35
Discussions of the Ohio State Dental Society........ 42
First District Dental Society of New York........ 50
SALMAGUNDI........ 53
In Memoriam........ 55

